# Retraction Note: Mindfulness-based randomized controlled trials led to brain structural changes: an anatomical likelihood meta-analysis

**DOI:** 10.1038/s41598-025-11069-9

**Published:** 2025-07-15

**Authors:** Savannah Siew, Junhong Yu

**Affiliations:** Psychology, School of Social Sciences, Nanyang Technological University, Singapore, Singapore

Retraction of: *Scientific Reports* 10.1038/s41598-023-45765-1, published online 27 October 2023

Editors have retracted this Article.

After publication of this study concerns were raised about the way the exclusion/inclusion criteria were applied. Specifically, four null-finding papers accounting for data from 40% of participants were excluded from the analyses, while other studies were included despite being done in very specific, nonrepresentative populations. In particular, the exclusion of the null papers means that the meta-analysis presented in the paper was not designed to test the stated hypothesis of whether mindfulness interventions have impact on brain morphology and cannot therefore support the conclusions of this Article.

Both Authors disagree with this retraction.

